# The combined impact of HLA and non-HLA mismatch between donors and recipients on kidney transplant survival: a genomic analysis in a prospective cohort

**DOI:** 10.1016/j.ebiom.2026.106420

**Published:** 2026-08-05

**Authors:** Michael Kammer, Andreas Heinzel, Stephen Shoebridge, Roman Reindl-Schwaighofer, Karin Hu, Hao Shan Chen, Alexander Kainz, Ismail Daoudi, Ana F. David, Gottfried Fischer, Brendan Keating, Matthias Niemann, Rainer Oberbauer, iGeneTRAiN consortium, Brian Piening, Alexa Dowdell, Mario Deng, Martin Cadeiras, Mengqi Zhang, Matthew B. Lanktree, Krzysztof Kiryluk, Francesca Zanoni, Sangho Lee, Ajay Israni, Brendan Keating, Rainer Oberbauer, Goran B. Klintmalm, Jacqueline O'Leary, Kim M. Olthoff, Elizabeth Rand, Abraham Shaked, John A. Belperio, Marc Buijsrogge, Jason D. Christie, Paul Corris, Andrew Fisher, Allan R. Glanville, Samuel Goldfarb, David Lederer, Keith C. Meyer, Henny G. Otten, Scott M. Palmer, David Wilkes, Nathan Pankratz, Pam Jacobson, Loren Gragert

**Affiliations:** a Division of Nephrology and Dialysis, Department of Medicine III, Medical University of Vienna, Vienna, Austria; b Institute of Clinical Biometrics, Center for Medical Data Science, Medical University of Vienna, Vienna, Austria; c Department of Transfusion Medicine and Cell Therapy, Medical University of Vienna, Vienna, Austria; d Department of Surgery, University of Pennsylvania, Philadelphia, PA, USA; e PIRCHE AG, Berlin, Germany; f Department of Nephrology and Medical Intensive Care, Charité Universitätsmedizin Berlin, Berlin, Germany

**Keywords:** Kidney transplantation, Histocompatibility, HLA matching, Graft failure risk

## Abstract

**Background:**

Kidney transplantation outcomes are strongly influenced by immunological compatibility between donor and recipient. While genetic mismatches in the human leukocyte antigen (HLA) region have long been recognised as key determinants of graft survival, increasing evidence, including our own previous work, suggests that non-HLA alloimmunity also plays a critical role.

**Methods:**

We sequenced exomes of deceased kidney donor and recipient pairs in the prospective kidney transplant cohort at the Vienna General Hospital, recruited between January 1, 2012, and June 15, 2023. Out of 1209 pairs, 1187 passed quality control for analysis. Non-HLA mismatch was computed by considering non-synonymous single nucleotide polymorphisms specifically encoding trans-cell membrane or secreted proteins in the kidney (nsSNP-tcmsk). Using adjusted Cox proportional hazards models, we replicated results from our earlier work in recipients with primary graft function after 90 days, and extended the analysis to the combination of nsSNP-tcmsk with eplet mismatch to assess their associations with graft loss in the full cohort.

**Findings:**

Of 20,421 human proteins, 2371 were considered for the nsSNP-tcmsk score. In our replication analysis we estimated for nsSNP-tcmsk a hazard ratio (HR) of 1.33 (95% CI 1.02–1.74) for graft loss per increase of one interquartile range. The nsSNP-tcmsk and eplet mismatch were uncorrelated (Spearman correlation coefficient 0.02, p = 0.47). A composite score of nsSNP-tcmsk and eplet mismatch was associated with graft loss with a HR of 1.76 (95% CI 1.20–2.57) corresponding to an absolute difference in 7-year restricted mean survival time between the first and fourth quartiles of 0.52 years (95% CI 0.16–0.87 years).

**Interpretation:**

The impact of non-HLA donor-recipient mismatch on transplant loss is of the same magnitude as established mismatch scores in the HLA region. Together, these scores may be further validated as guiding markers for the required strength of maintenance immunosuppression.

**Funding:**

Vienna Science and Technology Fund, NIH/NIAID.

**Research in context**

**Evidence before this study**

Human leukocyte antigen (HLA) matching between a kidney donor and the transplant recipient is routinely performed in the allocation process of many centres to reduce alloimmune responses and optimise graft survival. However, the compatibility in the alleles of the main 12 HLA genes explains only a moderate amount of variability in transplant outcomes. Recent findings from our institution and others suggest that donor-to-recipient mismatches outside the HLA region also contribute to alloimmunity and graft loss, but the estimated effect sizes differ between publications. Studies analysing the combination of HLA and non-HLA mismatch on a genomics scale are scarce.

In a structured literature search using a search string containing 13 kidney transplantation and 34 non-HLA related MeSH terms and keywords, a total of 1890 publications between 2019 and 2025 were identified in three different repositories (465 Medline, 1408 Embase, 17 PubMed Central). Twelve studies were deemed eligible for further investigation through the PICOTS framework (Population, Intervention, Comparator, Outcome, Time and Setting). Only four studies evaluated some type of genomic mismatch and graft loss, one of which was our previous publication (Lancet 2019, 393 (10174):910–917).

**Added value of this study**

This study corroborates the association of whole genomic mismatch between kidney donor and transplant recipient with graft loss. It is a conceptual and prospective replication of our previous analysis in a larger, contemporary cohort using new technologies. Specifically, this analysis elucidates the associations of modern molecular HLA mismatch scores (eplet) and of exome-wide non-HLA mismatch, as well as their combination, and quantifies the differences in graft survival time in absolute terms (i.e. years). Further, we applied state of the art sequencing technologies, which are now cheap and widely available, and developed a non-proprietary bioinformatics pipeline for the analysis of sequencing data in a large, prospective and fine granular cohort.

**Implications of all the available evidence**

The present study provides the basis for a change in risk estimation of histocompatibility in kidney transplantation. Genomic risk scores, which include structural HLA mismatches such as eplet mismatch in combination with the non-HLA mismatch, may be used to guide maintenance immunosuppression more precisely than by considering only HLA mismatches as done in current practice. Furthermore, the data on non-HLA alloimmunity may establish a framework to explain anti-HLA donor specific antibody (DSA) negative antibody mediated rejections seen in kidney transplantation, which were recently introduced in 2018 and continued in the Banff 2022 updated guidelines. Recent studies showed that the prognosis of cases with microvascular inflammation but no circulating anti-HLA DSAs is equally poor as those with HLA-DSAs. Thus, the present paper solidifies the evidence that immune responses against non-HLA alloantigens exist and are associated with premature graft loss. Unlike the anti-HLA DSA determination with ready-made solid phase technology, the individual donor to recipient mismatches on a genome-wide basis makes routine DSAs determination in the clinic currently impossible. Further studies will determine frequent and immunodominant non-HLA mismatches with the aim to generate a useable panel for the determination of non-HLA DSAs. The long history of HLA science and technologies in transplantation may facilitate these processes.

## Introduction

Kidney transplantation prolongs and restores the quality of life of persons with end-stage kidney failure. To achieve long-term graft patency lifelong immunosuppression reducing alloimmune processes is mandatory. However, the median half-life of deceased donor kidney transplants has not improved over the past decades to the same extent as short-term graft survival.^1^ More than half of the graft losses are attributable to chronic alloimmune events such as T-cell-mediated and antibody-mediated rejections (TCMR and ABMR), while the remaining dominant causes are infections and medical events, which may partly be side-effects of maintenance immunosuppression.^2^ This conundrum underscores the value of accurate risk stratification strategies, aiming to minimise immunosuppression without incurring heightened alloreactivity.

Human leukocyte antigen (HLA) matching is one such strategy, as donor-recipient (D/R) disparities in this highly polymorphic genomic region are well documented risk factors for graft loss.^3^ Over time, thousands of HLA class I and II alleles were reported to the HLA database,^4^ and the detection of anti-HLA donor-specific antibodies (DSAs) became standard in alloimmune risk evaluation. Modern histocompatibility scores use high-resolution two-field typing and some, like eplet matching, take into account predicted DSA-epitope interactions at the amino acid level. Still, the predictive capability and association of HLA mismatch with medium-term graft loss remain moderate. Even using molecular histocompatibility by predicting structural antibody epitopes, and taking major histocompatibility complex (MHC) T-cell epitope presentation into account, the c-statistic for 5-year death censored graft loss (DCGL) remains at 0.65.^5^

Since the roughly 200 HLA genes account for only 0.13% of the human genome, the genome-wide D/R antigen mismatch load is expected to be much larger than in the HLA region only,^6^ but the clinical relevance of minor histocompatibility antigens in kidney transplantation has long been disputed. Opelz and colleagues reported associations of graft loss with the presence of MHC class I chain-related gene A (MICA) antibodies in pre-sensitised recipients who were well matched for donor HLA,^7^ confirmed also by later studies.^8^ With increasing availability of high-throughput sequencing genome-wide mismatch burden became more widely studied,^9^ further suggesting that immune-mediated D/R interactions outside the HLA region contribute to graft loss. Several of the proteins encoded outside of the HLA region are polymorphic and may be subjected to genetic aberration during a lifetime.^10^

We showed previously, using genotyping with imputation, that in recipients with primary graft function after 90 days D/R mismatches outside the HLA region contribute to graft loss independently of HLA-mismatch.^11^ Additionally, we found significantly higher odds of specific non-HLA DSA development in pairs with mismatches outside the HLA region, specifically in cases with biopsy-confirmed microvascular inflammation. This aligns with the current Banff 2022 histopathological criteria for biopsy assessment, which classifies microvascular inflammation cases as ABMR even when no anti-HLA DSAs can be detected.^12^ Sablik and colleagues recently showed that the prognosis of such cases is equally poor as in DSA-positive ABMR.^13^

In this study, we address two aims. First, following the recent calls in The Lancet and other leading journals for updating and modernising genetic epidemiology studies we provide a replication of our previous findings in a larger, fine-granular, prospective cohort utilising the now widely available whole-exome sequencing (WES) technology for donors and recipients, as well as modern two-field HLA typing.^14^ Second, we extended our previous work using this new data and refined mismatch scoring by considering donor antigen localisation. This allowed us to evaluate the combined contribution of HLA and non-HLA mismatches to unambiguous outcomes such as graft and patient survival in a well-characterised clinical cohort.

## Methods

### Study design and participants

The Vienna Transplantation Cohort (VTC) is a prospective cohort comprising data from all 1679 adult kidney allograft recipients (≥18 years of age) transplanted at the General Hospital in Vienna from January 1, 2012, to June 15, 2023, and their respective donors. It reflects state-of-the-art post-transplant management, curating data from Eurotransplant, the Austrian Dialysis and Transplant Registry, and local hospital records, and collects clinical records as well as biobanked samples with almost no loss-to-follow-up. The end of follow-up for the present study was August 31, 2024.

For most D/R pairs WES was performed, given signed consent and the availability of recipient and donor DNA collected from samples taken at the time of transplantation. We excluded pairs with living donors (due to expected differences in genetic mismatch between related and un-related transplantations), recipients of multiple organs, and pairs with either sample failing sequencing quality control. We further excluded a small subset of 158 pairs (later assessed in a sensitivity analysis) with either donor or recipient having non-European genetic ancestry determined from WES because higher mismatch scores for mixed ancestries together with worse graft survival reported for some ancestries could falsely inflate the association between genetic mismatch and graft survival in our analysis. We defined a replication subcohort aligning to the population in our score development paper^11^ by restricting to pairs with ascertained primary graft function 90 days post-transplant. The initial development cohort had a small overlap with the VTC due to overlapping recruitment windows. As the VTC was planned as complete, prospective, biobanked cohort starting 2012 we still accepted these pairs (67, 7% of main analysis cohort) in the presented analyses since we updated their outcome data, used a more modern gene sequencing technology, and aimed at a more general target population with the present study. We assessed the impact on our main results in a sensitivity analysis by removing the transplant pairs already present in the earlier study.

The main outcome in our study was DCGL, defined as return to dialysis or re-transplantation. As secondary outcomes, we studied a composite of graft loss and recipient death, as well as TCMR and ABMR, both rescored exclusively from Banff score strings and DSA surveillance data adhering to Banff 2022 criteria (without consideration of other clinical findings). We administratively censored follow-up at 7.5 years due to large uncertainties for later, sparse event times. To elucidate the timing of associations we also analyse the main outcome with administrative censoring at 1, 3 and 5 years.

### Procedures and bioinformatics

Genomic DNA was extracted from blood samples of kidney transplant recipients and from either blood samples or spleen tissue of donors, depending on availability. A minimum of 2 μg of DNA was sent to Regeneron in 2D barcoded 0.5 ml matrix tubes (Thermo Fischer, cat. nr.: 3744BLU); of these 125 ng of DNA were processed with the Twist Comprehensive Exome (TCE) design. Libraries were sequenced on a NovaSeq XPlus from Illumina Inc. and reads were stored in per sample compressed reference-oriented alignment map (CRAM) files generated using the original quality functionally equivalent protocol available as a non-peer reviewed preprint.^15^

Variant calling for genomic mismatch analysis was performed using GATK version v4.6.0.0 (preprint, not peer-reviewed).^16^ Original sequencing reads were reconstructed from CRAM files and realigned against the Broad hg38 (V0) genome assembly using BwaSpark, following GATK best practices: duplicates were flagged using MarkDuplicatesSpark and base quality scores were adjusted using BQSRPipelineSpark using the reference genome corresponding *known_indels.vcf* and *dbsnp138.vcf* as known polymorphic sites to skip over. Calling of germline variants from pre-processed reads was carried out individually for each sample using HaplotypeCaller in genomic variant call format (gVCF) mode. Subsequently, information on sites within the Twist Comprehensive Exome target regions padded by 100 base pairs was imported from individual gVCF files into a common GenomicsDB using GenomicsDBImport and joint genotype calling was performed using GenotypeGVCFs with a minimum confidence threshold of 20. Variants in the resulting study level variant call format file were filtered for excess heterozygosity (p-value cutoff 3.4 × 10^−6^), a site depth of less than 50, and low recalibrated variant scores (sensitivity threshold of 99.7%) estimated from strand bias, normalised variant confidence, bias in position of reference- and alternate alleles within the supporting reads and bias in the quality score of reference and alternate alleles. In addition to variant filtering, genotype calls with a genotype quality score of less than 20 or read depth less than 10 were removed (no-call). Ensembl Variant Effect Predictor (VEP) version 113.0 was used to annotate the cohort level VCF file with variant consequences.^17^

### Genetic ancestry and identity-by-descent

Genotypes in unobserved regions were inferred through imputation using the exome sequencing data augmented with sequence information in backbone regions captured through Twist Biosciences’ “Diversity SNP Panel”. For ancestry estimation, SNPs were filtered to a minimum minor allele frequency (MAF) of 5% and maximum missingness of 10% and merged with HapMap3 using common sites. Principle components (PC) were calculated for HapMap samples and D/R samples were projected onto those PCs. Kernel density estimators (KDE) were trained on PC1 vs PC2 and PC3 vs PC4 from HapMap3 to assign D/R samples to HapMap3 populations (donors/recipients whose KDE assigned reference population was CEU or TSI were considered to be of European descent and included in the primary analysis). Identity-by-descent was estimated in the study dataset using plink version 1.9.^18^ The data set was restricted to SNPs on autosomes with a MAF of 10%, a maximum missingness of 5% and a minimum Hardy–Weinberg equilibrium exact test p-value of 0.000005. Variants in high linkage disequilibrium were removed using a window size of 50 and an *r*^*2*^ threshold of 0.2.

### Donor-to-recipient SNP mismatch

D/R SNP mismatch scores were calculated from bi-allelic SNPs on autosomes, outside of the HLA region (excluding chr6:28510120-33480577; GRCh38) captured directly by WES. The analysis was restricted to sites passing all filters. A D/R mismatch was defined on a per site basis as any D/R genotype constellation in which the donor carried an allele unknown to the recipient. Two versions of D/R SNP mismatches differing in sites/variants were considered: (1) to replicate the score from the original publication we used missense variants affecting transmembrane or secreted proteins (nsSNP-tms) and (2) to refine the score we considered only variants affecting transmembrane in cell membrane or secreted proteins, which are transcribed in the kidney (nsSNP-tcmsk). Transmembrane or secreted proteins were identified based on UniProt annotation (query: (ft_transmem:∗ OR cc_scl_term:SL-0243 OR keyword:KW-0812) AND organism_id:9606 AND reviewed:true). For nsSNP-tcmsk proteins were additionally required to be expressed in the kidney according to Human Protein Atlas transcriptome analysis (query: NOT tissue_category_rna:kidney; not detected) and, unless secreted, to be annotated with “Cell membrane” (Uniprot location: SL-0039).^19^ We provide the list of transcripts considered for nsSNP-tcmsk as Supplementary File. Ensembl Variant Effect Predictor (VEP) version 113.0 was used to annotate the cohort level VCF file with variant consequences.

### HLA antibody testing

Anti-HLA antibody screening was performed using LABScreen Mixed Class I and II assays (One Lambda, West Hills, CA, USA), with positive samples subsequently characterised by LABScreen Single Antigen HLA Class I and II assays (One Lambda) on a Luminex 200 or FLEXMAP 3D instrument. A positivity threshold of 1000 MFI was applied. To address potential prozone effects, sera were pre-treated with EDTA at a final concentration of 5 mM prior to testing. DSA assignment was based on donor HLA typings for HLA-A, -B, –C, -DRB1, -DRB3/4/5, -DQA1, -DQB1, -DPA1, and -DPB1, preferentially using two-field high-resolution typing; one-field or serological results were used when high-resolution data were unavailable. DSAs were defined as HLA antibodies directed against a mismatched donor HLA antigen.

### Computation of eplet mismatch

We used the HLAMatchmaker algorithm implemented in the provided Excel sheets from http://www.epitopes.net/downloads.html (downloaded 27.5.2025) to compute eplet mismatch per D/R pair.^20^ We used the Matchmaker Excel version 4 for A/B/C loci and version 3.1 for DR/DQ/DP loci (note that DP was not used here). In case a two-field HLA typing for a specific locus of an individual was unavailable, we used the imputation algorithm for high-resolution data from serotype level data as implemented on the PIRCHE AG server.^21^ Each possible high-resolution typing arising from the low-resolution input data was then used in the Matchmaker Excel sheets, and the resulting eplet mismatches weighted according to the weights provided by the imputation algorithm to obtain the final eplet score.

### Composite score of HLA and non-HLA mismatch

To derive a single mismatch score representing overall D/R mismatch we created a rank-based composite of eplet mismatch for the A/B/C/DR/DQ loci (for details on the computation see Supplementary Methods) and nsSNP-tcmsk. By computing their respective empirical cumulative distributions (ECDF) we normalised both mismatch scores to the range [0, 1] and summed them to weigh their contributions equally. The resulting composite score had range [0, 2], with zero representing D/R-pairs with the lowest combined HLA and non-HLA mismatch in our cohort, two representing pairs with the highest combined mismatch, and unity representing pairs with “median” mismatch.

### Statistical analysis

Cohort characteristics were described by medians and interquartile ranges (IQR), or absolute and relative frequencies for continuous and discrete variables, respectively. We employed Kaplan–Meier analyses to describe univariable associations of the mismatch scores stratified by their respective medians with DCGL, quantifying differences between strata using log-rank tests. Correlations between mismatch scores were evaluated by Spearman's correlation coefficient. Our analysis proceeded in two steps. First, we confirmed the adjusted associations of nsSNP-tms and nsSNP-tcmsk with DCGL in the replication subcohort, after which we focused on the refined nsSNP-tcmsk score. Second, we studied the combined association of HLA mismatch burden via eplet mismatch and non-HLA mismatch burden via nsSNP-tcmsk in the main analysis cohort. In both analysis steps, we used multivariable Cox proportional hazards models, each comprising a non-HLA score and eplet mismatch, or their composite score. All models were adjusted for genetic relatedness through Pi-hat. Based on clinical considerations the models further comprised the Kidney Donor Profile Index (KDPI), donor sex, presence of preformed DSAs and cold ischaemia time. To compute the KDPI in our cohort we followed the algorithm in the original publication as implemented in the “kidney.epi” R package version 1.4.^22^^,^^23^ Besides the donor covariates, we used the year 2018 for the US Organ Procurement and Transplantation Network mapping table (any other mapping from 2014 to 2024 leaves the KDPI virtually unchanged), as well as the scaling factor. To account for the dependence created between transplantations when a single donor's two kidneys were transplanted to different recipients, we used robust covariance estimators to ensure correct statistical inference for hazard ratios. All mismatch scores were divided by their IQR for comparability. To assess proportionality we tested and plotted Schoenfeld residuals against each variable. For the fitted Cox models in the main analysis cohort we obtained stratified adjusted graft survival curves for the mismatch scores through direct regression standardisation. First, for each level of the stratification factor (e.g. the quartiles of the composite score) a copy of the dataset was created in which the stratification factor was set to this specific level (e.g. “first quartile”) for all recipients. Second, the fitted model was used to obtain predicted survival probabilities at each event time for each recipient in all copies of the dataset. Third, these survival probabilities were averaged at each event time over all recipients for each stratum separately. These adjusted survival curves were also used to compute restricted mean survival times (RMST) as the area under the adjusted survival curves for each level of a stratification factor until a pre-specified time point t. RMSTs provide absolute estimates of survival times and can be interpreted as the expected graft survival time per stratum of the cohort observed up to a specified timepoint.^24^ Confidence intervals (CI) for RMSTs were obtained by bootstrap resampling. To investigate the linearity of the mismatch scores (eplets, nsSNP-tcmsk and composite score) we used penalised splines with four degrees of freedom and likelihood ratio tests, while the other variables remained linear in the model. Our main conclusions were based on the models using scores as continuous variables. Score categorisation served exclusively for additional descriptive visualisation via univariable Kaplan–Meier curves, adjusted survival curves, and RMST estimates across clinically relevant groups. For the secondary outcomes, the impact of recipient death on the results was assessed by a Cox proportional hazards model for overall graft survival as the outcome variable and the same adjustment as for the DCGL analysis, as well as by a cumulative incidence function disambiguating graft loss and recipient deaths. For TCMR and ABMR similar Kaplan–Meier analyses and Cox proportional hazards model were fitted as for the DCGL analysis. Two-sided p-values less than 0.05 were considered indicating statistical significance with no adjustment for multiple testing. Due to the very low number of missing values D/R pairs with missing data were removed before analysis. All statistical analyses were performed in the R software version 4.4 (using packages “survival” version 3.8.3, and “adjustedCurves” version 0.11.3).

### Sensitivity and generalisability

To investigate the sensitivity of our results to the occurrence of de-novo DSA (dnDSA) after transplantation as potential pathway affecting the study outcomes, we added a time-dependent binary variable encoding a positive dnDSA diagnosis to our analysis models. To assess generalisability, we re-fitted the main analysis models in a cohort including transplants involving non-European recipients or donors. Further, since RMSTs critically rely on the proportionality assumption we corroborated the results using estimation based on pseudo-values.^25^ To analyse the estimated pseudo-values based on the Kaplan–Meier method we used generalised estimating equations (GEE) comprising the same stratification factors and covariates as the Cox proportional hazards models used for the adjusted survival curves. We obtained RMSTs for the different mismatch score strata as predictions from the fitted GEE models when setting the level of the stratification factor to a specific value for all transplantation pairs. This was done for the different pre-specified RMST horizons separately. The R package “eventglm” version 1.4.5 was used to implement this analysis. We assessed the stability of the ranking based composite score through resampling as follows. We drew 500 samples with replacement from the main analysis cohort, recomputed the composite score based on the ECDF separately in each resample, and re-fitted the main analysis model on this data to assess if the estimated hazard ratio for the composite score was stable across resamples. As alternative to the quartile-based mismatch score strata, we also provide a simpler analysis based on stratifying eplet mismatch and nsSNP-tcmsk by their respective medians and combining the resulting strata.

### Ethics statement

The study was conducted in accordance with the principles of the 2024 Declaration of Helsinki, and approved by the institutional review board of the Medical University of Vienna (EK Nr 2011/267, 1040/2019 and 2041/2024). All living participants gave written informed consent before genomic sequencing. In Austria, deceased donor organ donation is governed by the national organ transplantation law enforcing an opt-out system i.e. informed consent was waived.

### Role of the funding source

The funders had no role in study design, data collection and analysis, the decision to publish or writing the manuscript. The corresponding author had full access to all of the study data and takes responsibility for the integrity of the data and its analysis.

## Results

### Cohort description

**Fig. 1:**
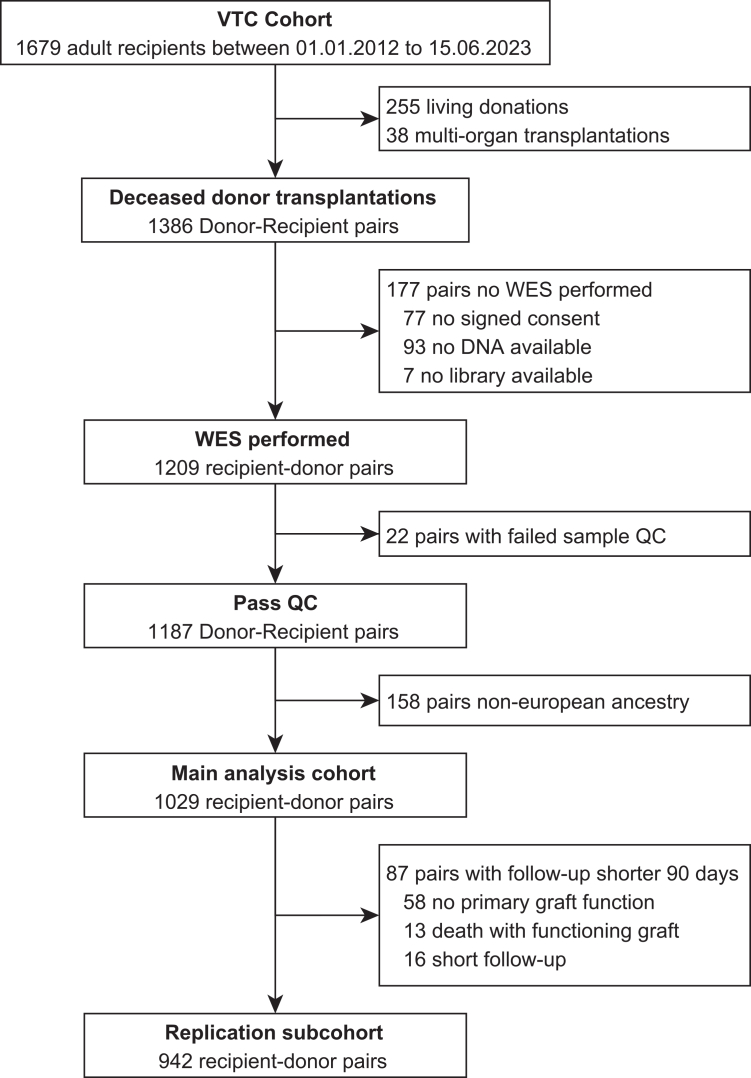
**Study design.** Flowchart of donor/recipient pair inclusion.

In total 1029 D/R pairs were analysed (Fig. 1, Table 1, Supplementary Table S1), comprising 751 distinct deceased donors of whom 278 (37%) donated both kidneys. The median observed follow-up was 4.3 years (IQR 1.9–6.9) during which 160 (16%) allograft losses were observed and 302 (29%) recipients died. Only 376 (37%) of the recipients were female, reflecting the worldwide higher prevalence of kidney disease in men.

**Table 1:** Transplant characteristics.

Characteristic	Measure (n = 1029)	Missing
Recipient age, years	57 (48, 65)	–
Recipient sex		–
Female	376 (37%)	
Male	653 (63%)	
Dialysis vintage, years	3.1 (2.1, 4.5)	1 (<1%)
First transplantation, %	832 (81%)	–
Donor age, years	57 (46, 67)	–
Donor sex		–
Female	466 (45%)	
Male	563 (55%)	
Kidney donor profile index	71 (43, 90)	7 (<1%)
DCD Donor	144 (14%)	1 (<1%)
Cold ischaemia time, hours	11.5 (7.5, 15.3)	–
Panel reactive antibodies, %	0 (0, 0)	3 (<1%)
Preformed donor HLA specific antibodies	140 (14%)	1 (<1%)
HLA serotype mismatch (HLA-A, HLA-B, HLA-DR)		4 (<1%)
0	61 (6%)	
1	61 (6%)	
2	211 (21%)	
3	321 (31%)	
4	251 (24%)	
5	86 (8%)	
6	34 (3%)	
Eplet mismatch	28 (19, 38)	1 (<1%)
Observed follow-up, years	4.3 (1.9, 6.9)	–
Biopsy-confirmed TCMR (full study period)	96 (9%)	–
Biopsy-confirmed ABMR^a^ (full study period)	128 (12%)	–
De-novo donor specific antibodies (full study period)	128 (12%)	–
Class I^b^	51 (5%)	
Class II^b^	98 (10%)	
Observed graft failures (full study period)	160 (16%)	–
Observed deaths (full study period)	302 (29%)	–

Data for 1029 donor-recipient pairs are reported by median (interquartile range) or frequency (percent). DCD = Donation after Circulatory Determination of Death.

a Including microvascular inflammation.

b Only considering the first diagnosis after transplantation.

The median number of non-synonymous SNP mismatches in transmembrane and secreted proteins (nsSNP-tms) was 1768 (IQR 1727–1806), reduced to 587 (IQR 569–605) after restriction to proteins located in cell membranes and transcribed in the kidney (nsSNP-tcmsk, Supplementary Figures S1 and S2). While both non-HLA scores were moderately correlated (Spearman correlation $r_{s}$ 0.55, p < 0.001), neither correlated with eplet mismatch ($r_{s}$ 0.01, p = 0.715 and $r_{s}$ 0.02, p = 0.476 for nsSNP-tms and nsSNP-tcmsk, respectively, Supplementary Figure S3). The nsSNP-tcmsk score was only weakly correlated with overall genetic D/R relatedness ($r_{s}$ −0.1, p ≤ 0.001, Supplementary Figure S4).

### Replication of non-HLA mismatch score outcome association

**Fig. 2:**
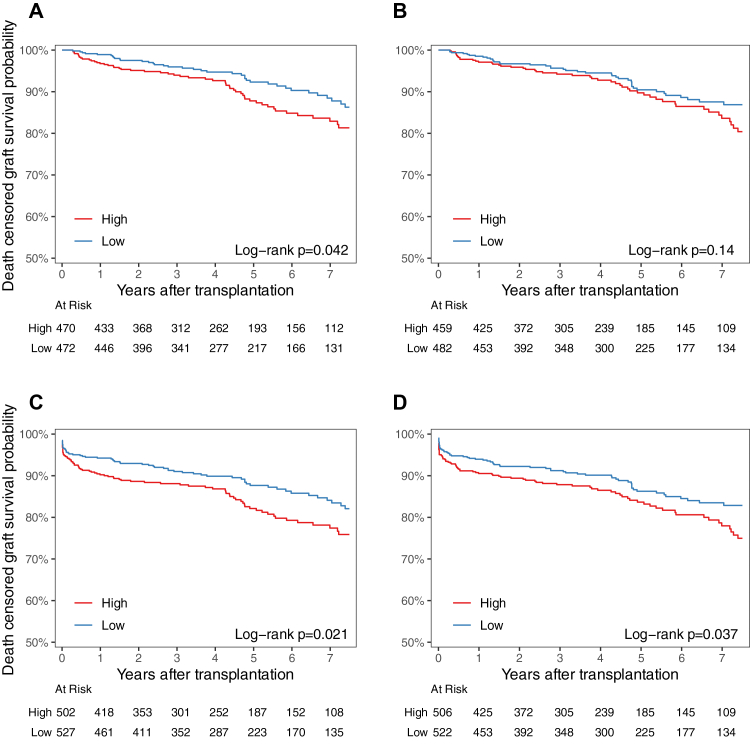
**Kaplan–Meier plots of death-censored graft survival, stratified by either nsSNP-tcmsk mismatch or HLA eplet mismatch in replication and main analysis cohorts.** The upper row shows Kaplan–Meier plots for death-censored graft survival for the replication subcohort (excluding the first 90 days), stratified by the median of nsSNP-tcmsk mismatch in this subcohort (median 586) in panel A, and HLA eplet mismatch (median 28) in panel B. The lower row shows results for the main analysis cohort, stratified by the median of nsSNP-tcmsk mismatch in this cohort (median 587) in panel C, and HLA eplet mismatch (median 28) in panel D. In all panels, “High” denotes recipients with higher than sample median mismatch for the corresponding score, “Low” those with mismatch lower than the sample median. Differences between the survival curves are reported by the p-values from log-rank tests.

The replication subcohort of 942 D/R pairs with primary graft function confirmed at 90 days post-transplant had similar characteristics as the main analysis cohort (Supplementary Table S2). A multivariable Cox model corroborated the significant association of our initially developed nsSNP-tms score with DCGL (Supplementary Table S3), with each increase by a unit of one IQR having a HR of 1.31 (95% CI 1.03–1.67, p = 0.028). Further, the refined score nsSNP-tcmsk had a similar HR of 1.33 (95% CI 1.02–1.74, p = 0.033, Table 2). After adjustment eplet mismatch was not significantly associated with graft loss in either model, but its estimated HR and CI were comparable to that of our non-HLA scores (full models in Supplementary Table S3). This was consistent with the univariable Kaplan–Meier analysis showing a significant association between mismatch load and DCGL in the replication subcohort stratified by the median nsSNP-tcmsk score, but not by eplet mismatch (Fig. 2, top row).

**Table 2:** Multivariable Cox proportional hazards models for death-censored graft loss.

	Replication subcohort		Main analysis cohort	
Separate HLA + non-HLA				
nsSNP-tcmsk	1.33 (1.02, 1.74) p = 0.033	Combined p = 0.045	1.19 (0.97, 1.47) p = 0.096	Combined p = 0.034
Eplet mismatch	1.19 (0.90, 1.57) p = 0.227		1.25 (0.99, 1.57) p = 0.062	
Composite score model				
Composite score	1.91 (1.11, 3.29) p = 0.020		1.76 (1.20, 2.57) p = 0.004	

The four cells of the table correspond to four different Cox models, differing in the cohort they were fitted in (columns) and how HLA and non-HLA scores entered the models (separately, top row, or as composite score, bottom row). Data are shown as hazard ratios (95% confidence interval) and p-values. Combined p-values were derived via likelihood ratio tests when dropping both coefficients from the model. All models were additionally adjusted for KDPI, donor age, donor sex, presence of preformed DSAs, cold ischaemia time and Pi-hat, with robust Sandwich covariance estimators. nsSNP-tcmsk and eplet mismatch were standardised by interquartile ranges. For all models 8 observations were removed due to missing data; 89 and 147 death-censored graft-losses within 7.5 years after transplantation were used in the models for the replication subcohort and the main analysis cohorts, respectively.

### Main analysis of HLA and Non-HLA mismatch scores

**Fig. 3:**
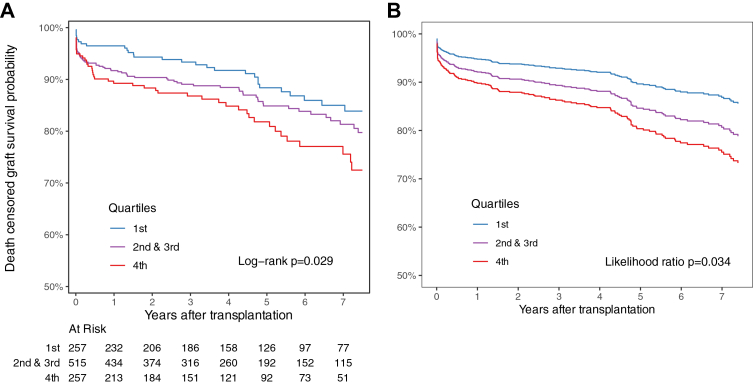
**Kaplan–Meier and adjusted curves for death-censored graft survival for the composite score.** Left panel A shows a Kaplan–Meier curve stratified by quartiles of the composite score of HLA eplet mismatch and nsSNP-tcmsk (the middle second and third quartiles were merged for clarity). The overall difference between the survival curves is reported by the p-value from a log-rank test. Right panel B shows corresponding adjusted survival curves computed using a Cox proportional hazards model adjusted for donor age, KDPI, donor sex, presence of preformed DSAs, cold ischaemia time and Pi-hat. The overall difference between the survival curves is based on the coefficient of the composite score in the main Cox proportional hazards model, hence we report the p-value from the corresponding likelihood ratio test as given in Table 2.

In the main analysis cohort, univariable Kaplan–Meier analyses showed that the discrimination between the strata defined by median nsSNP-tcmsk and eplet mismatch were both significant (Fig. 2, bottom row). In an adjusted Cox model neither nsSNP-tcmsk nor eplet mismatch were significantly associated with DCGL individually, however a combined likelihood ratio test indicated that dropping both resulted in a significantly worse model fit (Table 2). The associations of the two scores were largely independent. When one score was removed from the model, the estimated association for the remaining score was unchanged. Further, the composite score giving equal weight to nsSNP-tcmsk and eplet mismatch was significantly associated with DCGL (HR 1.76, 95% CI 1.20–2.57, p = 0.004, Table 2), where one unit of the score corresponds to the step from lowest mismatch to median mismatch, and from median to highest mismatch (full model in Supplementary Table S4). Based on the adjusted survival curves (Fig. 3) the RMSTs showed a significant difference of 0.52 years (95% CI 0.16–0.87) over 7 years follow-up between the highest mismatch stratum (fourth quartile of composite score) and the lowest (first quartile, Table 3). Model diagnostics based on Schoenfeld residuals did not indicate problems with the proportional hazards assumption in any of the models (Supplementary Table S5, Supplementary Figure S5). The investigation of non-linearities using splines also did not reveal any potential misspecification (Supplementary Figure S6). The association of the composite score with DCGL remained stable when the DCGL outcome was administratively censored earlier at 1, 3, and 5 years, albeit with larger uncertainty due to fewer events (Supplementary Table S6). The sensitivity analysis removing 67 transplant pairs also present in the development cohort showed almost identical results for the composite score as the main analysis (Supplementary Table S7). Altogether, this analysis supports the benefits of using both HLA and non-HLA scores.

**Table 3:** Restricted mean survival times for composite score of nsSNP-tcmsk and eplet mismatch in main analysis cohort.

Year	High mismatch	Low mismatch	Difference
1	0.92 (0.89, 0.94)	0.96 (0.94, 0.97)	0.04 (0.01, 0.07)
3	2.68 (2.58, 2.77)	2.83 (2.77, 2.90)	0.16 (0.04, 0.28)
5	4.36 (4.18, 4.54)	4.67 (4.55, 4.79)	0.31 (0.09, 0.53)
7	5.92 (5.63, 6.21)	6.43 (6.23, 6.64)	0.52 (0.16, 0.87)

Restricted mean survival times are given by point estimates (95% confidence intervals derived by bootstrap) and represent times in years. Strata are defined by the composite score of nsSNP-tcmsk and eplet mismatch (high mismatch given by 4th quartile of the composite score, low mismatch as the 1st). The values were estimated using direct regression standardisation with a Cox proportional hazards model adjusted for donor age, KDPI, donor sex, presence of preformed DSAs, cold ischaemia time and Pi-hat.

### Secondary outcomes and sensitivity analyses

Analysis of the secondary outcome combining recipient death and graft loss resulted in a smaller HR for nsSNP-tcmsk and the composite score (composite score HR 1.47, 95% CI 1.15–1.87, p = 0.003, Supplementary Table S8), indicating a stronger association with graft loss than with recipient death. The cumulative incidence of death was around 30% at 7.5 years of follow-up (Supplementary Figure S7). The analysis of TCMR and ABMR showed a reduced association of the composite score with these outcomes (Supplementary Table S9, Supplementary Figure S8).

The results for nsSNP-tcmsk and the composite score were robust in our sensitivity analyses. Adding a time-dependent variable for the formation of dnDSA expectedly resulted in a strong association of dnDSA with graft failure, thereby weakening the associations of the mismatch scores with graft failure as dnDSA may partly mediate such associations. However, the association of our composite score with the outcome remained significant with a roughly comparable point estimate as in the main analysis (HR 1.56, 95% CI 1.06–2.31, p = 0.026, Supplementary Table S10). RMSTs for the simplified combination of eplet mismatch and nsSNP-tcmsk by median strata were comparable to that of the rank-based composite score (Supplementary Figure S9, Supplementary Table S11). In a more general cohort including the 158 D/R-pairs with genetic non-European ancestry the nsSNP-tcmsk and composite scores remained significantly associated with DCGL (HR 1.12, 95% CI 1.02–1.24, p = 0.024 and HR 1.72, 95% CI 1.22–2.41, p = 0.002 respectively, Supplementary Table S12). The results for RMSTs using pseudo-values were almost identical to those obtained via the Cox models (Supplementary Table S13). The bootstrap procedure for the composite score showed that the estimate was stable (Supplementary Figure S10).

## Discussion

In this study, we conceptually replicated our previous paper that showed the association of D/R mismatches in genomic regions outside the HLA system with kidney graft failure.^11^ This aligns with the need for replication to ensure the reliability of scientific evidence, as highlighted by a *Nature* survey of over 1500 researchers that revealed a serious reproducibility crisis.^26^ We further refined our previous non-HLA score to better target likely immunogenic variants transcribed in the kidney itself and used an independent contemporary and larger cohort of deceased donor kidney transplant recipients with state-of-the-art genomics analyses. Lastly, we derived a composite of HLA and non-HLA mismatches that showed good discriminative abilities in a cohort reflecting the clinical population at our transplant centre. Its association was largely independent of other known predictors of graft survival, such as KDPI and the presence of preformed DSAs.

Fully external validation of our earlier results on non-HLA mismatch was further provided by Markkinen et al. in a kidney transplant cohort from Helsinki of 1025 D/R pairs.^27^ Due to a small number of 68 graft losses, but 199 rejections during a median observation period of three years, only the rejection outcome reached statistical significance. Most other studies that investigated non-HLA D/R mismatch evaluated rejections or dnDSA occurrence rather than the clinically most relevant outcomes graft loss and death.^28^ Furthermore, contemporary cohorts with modern immunosuppression under tight surveillance exhibit a generally lower rate of graft loss and thus the difference in graft survival between well and poorly matched donors and recipients becomes smaller.^29^^,^^30^

In solid organ transplantation, some groups studied the cumulative effect of many non-HLA mismatches, while others focused on specific variants associated with graft loss. Demir and colleagues recently showed that HLA class II molecular mismatches defined by eplets and indirect T cell epitopes but not HLA evolutionary divergence are independently associated with ABMR suggesting limited value of HLA evolutionary divergence for risk stratification on the population level.^29^ The higher mismatch load per IQR for non-HLA mismatch makes it difficult to elucidate if individual specific mismatches are immunodominant. Studies on H–Y mismatches between donor and recipients found only a weak association with graft survival adjusted for weight differences. However, in haematopoietic stem cell transplantation (HSCT) it has been shown that male recipients of female donor stem cells exhibit a higher risk of GVHD.^31^ Consequently, the frequency of female donor to male recipient HSCTs declined over the last decades. Other minor histocompatibility antigen mismatches have been described in HLA-identical siblings, as sets of peptides binding HLA allotypes in the recipient and evoking immune responses.^32^ Cao et al. developed and validated a polygenic risk score using data from 748 DeKAF and 352 Gen-03 living donor kidney transplant recipients with unspecified rejection.^33^ Pineda et al. used an exome sequencing approach and found a significantly higher number of mismatched non-HLA variants in a group of 17 cases with ABMR but not in seven TCMR and seven controls.^34^

Regarding specific variants, Steers and colleagues found that an intronic alteration in the LIMS1 locus represented by the singe nucleotide variant rs893403 that tags a copy number variation showed a statistically significant association with allograft rejection even after adjustment.^35^ This finding was validated in multiple other cohorts, e.g. Sun et al.,^36^ and functionally validated. Similar associations with graft loss and rejection were reported for MICA.^8^^,^^37^

Zhang et al. demonstrated a significant association between renal allograft survival and the proportion of genome-shared identity-by-descent independent of HLA mismatches between D/R pairs of similar ancestry.^38^ Among the 224 Caucasian transplant D/R pairs, a strong association was observed (HR 0.03, 95% CI: 0.001–0.79), which highlights the importance of individual ancestry even within the same racial group.

In contrast to our work based on exonic, non-synonymous SNPs, Zhang and Cao et al. studied the association of genome-wide SNP mismatch with graft rejection/loss including intronic regions. Furthermore, Cao's polygenetic risk score was derived and validated in live donor transplants.

As any observational genetic epidemiological study, our analysis has limitations. As such, it cannot establish causality, but solid exome-wide associational studies help to set the stage for future functional, mechanistic and therapeutic studies.^39^^,^^40^ In particular, we did not conduct a formal immunogenicity analysis (e.g. HLA peptide presentation or binding prediction) in this study. Here we focused on genomic donor–recipient mismatch burden and clinical outcomes; hence, the incorporation of immunogenicity modelling represents an important next step and will be addressed in future work. As potential refinement of the proposed scoring system HLA presentability can be considered by filtering mismatches based on the (predicted) affinity towards HLA molecules.^9^^,^^41^ Another intrinsic issue is that the effects of individual risk factors for graft loss are generally small and thus require large sample sizes to be identified. In addition, the uncertainty about tissue specific gene expression under disease, the imbalance between the number of potential genetic variants, the low frequency of rare but potentially important variants and the different distribution of most of the SNPs in different ethnicity/race groups, makes reproduction of findings difficult. This was mitigated by validating the findings of our previous paper in the same clinical setting in a prospectively and precisely curated cohort. Further strengths of the present study include the excellent data quality as evidenced in the very low rate of missingness of data and virtually no loss to follow up. While we could replicate the findings of our initial study published some years earlier,^11^ to further generalise these findings more diverse genetic backgrounds need to be analysed.

In this study, we validated and refined our previous findings in a contemporary cohort. The contribution of non-HLA D/R mismatches to transplant loss was of a similar magnitude as established mismatch scores in the HLA region. Together, these findings suggest that combined mismatch scores warrant further investigation as guiding markers for the prescription and dosing of maintenance immunosuppression.

## Contributors

MK, AH, RR-S, and RO conceived and designed the analysis. BK contributed to the conceptualisation of the study. RR-S, AH, AK, KH, HSC, and ID collected the phenotype and genetic data. GF contributed and analysed HLA data. KH, HSC, AH, RR-S were responsible for sample handling for sequencing. BK contributed to whole exome sequencing. AH and SS conducted the genetics and bioinformatics analysis. MN contributed to bioinformatics. MK, AH, SS, AD, and RO did the statistical analysis and interpreted the data. MK, AH, and RO wrote the draft of the report. All authors revised the report for important intellectual content. All authors have read and approved the final version of the manuscript. MK, AH, and RO had full access to the study data and verified its accuracy. RO is the guarantor of this work and, as such takes responsibility for the integrity of the reported data analysis. The iGeneTRAiN consortium contributed to whole exome sequencing and provided the foundation for the conceptualisation of the study.

## Data sharing statement

Individual level data can be made available on a collaborative basis upon request to rainer.oberbauer@meduniwien.ac.at following institutional review board approval and in accordance with the General Data Protection Regulation. The code used to produce the figures and tables of this paper is made available as supplemental data to this work, hosted on Zenodo (https://doi.org/10.5281/zenodo.20719360).

## Declaration of interests

MN is an employee and holder of phantom stock options of PIRCHE AG, which develops and operates the TxPredictor web service. PIRCHE AG has filed a patent application on matching of surface-accessible amino acid mismatches, and MN is listed as the inventor on this patent. BK declares support by several grants (NIH R01AI144522, NIH U19AI191396, NIH U01-AI163072). The remaining authors declare no potential conflicts of interest.

## Appendix A Supplementary data

Supplementary Material

Study Group
