# Supplementary material for: The combined impact of HLA and non-HLA mismatch between donors and recipients on kidney transplant survival: a genomic analysis in a prospective cohort
Source: eBioMedicine. 2026 Aug 5;131:106420. doi: 10.1016/j.ebiom.2026.106420 (PMC13471318; doi:10.1016/j.ebiom.2026.106420)
Supplement: Supplementary file 1 [file mmc1.docx]

**Supplement to:
The combined impact of HLA and non-HLA mismatch between donors and recipients on kidney transplant survival: a genomic analysis in a prospective cohort**

Michael Kammer PhD^1,2*^, Andreas Heinzel MSc^1*^, Stephen Shoebridge MSc^1^, Roman Reindl-Schwaighofer MD^1^, Karin Hu MSc^1^, Hao Shan Chen MSc^1^, Alexander Kainz PhD^1^, Ismail Daoudi MD^1^, Ana F. David PhD^1^, Gottfried Fischer MD^3^, Brendan Keating DPhil^4^, Matthias Niemann MSc^5,6^, Rainer Oberbauer PhD^1^ on behalf of the iGeneTRAiN consortium^7^

1 Medical University of Vienna, Department of Medicine III, Division of Nephrology and Dialysis, Vienna, Austria

2 Medical University of Vienna, Center for Medical Data Science, Institute of Clinical Biometrics, Vienna, Austria

3 Medical University of Vienna, Department of Transfusion Medicine and Cell Therapy, Vienna, Austria

4 University of Pennsylvania, Department of Surgery, Philadelphia, PA, USA

5 PIRCHE AG, Berlin, Germany

6 Department of Nephrology and Medical Intensive Care, Charité Universitätsmedizin Berlin, Berlin, Germany

7 For the iGeneTRAiN consortium see http://iGeneTRAiN.org

*Contributed equally

Correspondence to:

Prof. Rainer Oberbauer

Department of Nephrology and Dialysis, Internal Medicine III, Medical University of Vienna

Währinger Gürtel 18-20, 1090 Vienna, AUSTRIA

+43 (0)1 40400-43900

rainer.oberbauer@meduniwien.ac.at

Contents

[Supplementary Tables	3](#_Toc233795010)

[Supplementary Table S 1: Transplant characteristics for main analysis cohort, disaggregated by donor and recipient sex.	3](#_Toc233795011)

[Supplementary Table S 2: Transplant characteristics for replication, main analysis and full VTC cohorts.	4](#_Toc233795012)

[Supplementary Table S 3: Replication subcohort models.	5](#_Toc233795013)

[Supplementary Table S 4: Main analysis models.	6](#_Toc233795014)

[Supplementary Table S 5: Main analysis model diagnostics - Tests for Schoenfeld residuals.	7](#_Toc233795015)

[Supplementary Table S 6: Main model for composite score with different administrative censoring times.	8](#_Toc233795016)

[Supplementary Table S 7: Sensitivity analysis removing 67 transplants present in earlier study.	9](#_Toc233795017)

[Supplementary Table S 8: Generalisability of main models to all-cause graft loss in main analysis cohort.	10](#_Toc233795018)

[Supplementary Table S 9: Cox proportional hazard model for secondary TCMR and ABMR outcomes in main analysis cohort.	11](#_Toc233795019)

[Supplementary Table S 10: Main model including time-dependent covariate for the formation of de-novo DSAs.	12](#_Toc233795020)

[Supplementary Table S 11: Restricted mean survival times for strata defined by median scores of nsSNP-tcmsk and eplet mismatch in main analysis cohort.	13](#_Toc233795021)

[Supplementary Table S 12: Generalisability of main model to cohort including transplant pairs with non-European ancestry.	14](#_Toc233795022)

[Supplementary Table S 13: Sensitivity analysis for restricted mean survival time through alternative computation based on pseudo-values in main analysis cohort.	15](#_Toc233795023)

[Supplementary Figures	16](#_Toc233795024)

[Supplementary Figure S 1: Flowchart of genomic variant inclusion based on our main analysis cohort.	16](#_Toc233795025)

[Supplementary Figure S 2: Distributions of mismatch scores in main analysis cohort.	17](#_Toc233795026)

[Supplementary Figure S 3: Scatter plots of HLA eplet mismatch and non-HLA mismatch scores in the main analysis cohort.	18](#_Toc233795027)

[Supplementary Figure S 4: Scatter plot of nsSNP-tcmsk mismatch score and genomic distance (Pi-hat, Identity by descent) in the main analysis cohort.	19](#_Toc233795028)

[Supplementary Figure S 5: Main model diagnostics - Plots for Schoenfeld residuals.	20](#_Toc233795029)

[Supplementary Figure S 6: Assessment of the linearity assumption for mismatch scores using penalised splines.	21](#_Toc233795030)

[Supplementary Figure S 7: Cumulative incidence functions for graft loss and competing event death in the main analysis cohort.	22](#_Toc233795031)

[Supplementary Figure S 8: Univariable Kaplan-Meier plots of TCMR and ABMR after transplantation stratified by quartiles of the composite score in the main analysis cohort.	23](#_Toc233795032)

[Supplementary Figure S 9: Univariable Kaplan-Meier and adjusted survival curves for death-censored graft survival using the combination of HLA eplet mismatch and nsSNP-tcmsk by median strata in the main analysis cohort.	24](#_Toc233795033)

[Supplementary Figure S 10: Assessment of stability of hazard ratio estimate of composite score via bootstrap in the main analysis cohort.	25](#_Toc233795034)

# Supplementary Tables

## Supplementary Table S 1: Transplant characteristics for main analysis cohort, disaggregated by donor and recipient sex.

|  | **Female recipients,  Female donors** | | **Female recipients,  Male donors** | | **Male recipients,**  **Female donors** | | | **Male recipients,  Male donors** | |
| --- | --- | --- | --- | --- | --- | --- | --- | --- | --- |
| **Characteristic** | **Measure  (n = 166)** | **Missing** | **Measure  (n = 210)** | **Missing** | | **Measure  (n = 300)** | **Missing** | **Measure  (n = 353)** | **Missing** |
| Recipient age, years | 58 (49, 65) | - | 55 (46, 63) | - | | 60 (49, 67) | - | 57 (48, 65) | - |
| Recipient sex |  | - |  | - | |  | - |  | - |
| Female | 166 (100%) |  | 210 (100%) |  | | - |  | - |  |
| Male | - |  | - |  | | 300 (100%) |  | 353 (100%) |  |
| Dialysis vintage, years | 2.8 (1.8, 4.3) | - | 3.3 (1.9, 4.8) | 1 (<1%) | | 3.1  (2.0, 4.4) | - | 3.2  (2.2, 4.4) | - |
| First transplantation, % | 129 (78%) | - | 158 (75%) | - | | 252 (84%) | - | 293 (83%) | - |
| Donor age, years | 59 (48, 68) | - | 54 (46, 65) | - | | 60 (49, 70) | - | 54 (42, 66) | - |
| Donor sex |  | - |  | - | |  | - |  | - |
| Female | 166 (100%) |  | - |  | | 300 (100%) |  | - |  |
| Male | - |  | 210 (100%) |  | | - |  | 353 (100%) |  |
| Kidney donor profile index | 74 (51, 91) | - | 64 (41, 85) | 1 (<1%) | | 82 (56, 94) | - | 64 (36, 86) | 6 (2%) |
| DCD Donor | 19 (11%) | - | 32 (15%) | 1 (<1%) | | 32 (11%) | - | 61 (17%) | - |
| Cold ischemia time, hours | 11.5  (7.9, 15.4) | - | 11.2  (7.0, 15.1) | - | | 11.8  (7.9, 15.4) | - | 11.4  (7.3, 15.1) | - |
| Panel reactive antibodies, % | 0 (0, 0) | - | 0 (0, 0) | - | | 0 (0, 0) | 1 (<1%) | 0 (0, 0) | 2 (<1%) |
| Preformed donor HLA specific antibodies | 38 (23%) | - | 43 (20%) | - | | 24 (8%) | - | 35 (10%) | - |
| HLA serotype mismatch  (HLA-A, HLA-B, HLA-DR) |  | 2 (1%) |  | - | |  | - |  | 2 (<1%) |
| 0 | 11 (7%) |  | 17 (8%) |  | | 16 (5%) |  | 17 (5%) |  |
| 1 | 9 (6%) |  | 17 (8%) |  | | 18 (6%) |  | 17 (5%) |  |
| 2 | 34 (21%) |  | 38 (18%) |  | | 62 (21%) |  | 77 (22%) |  |
| 3 | 50 (30%) |  | 70 (33%) |  | | 82 (27%) |  | 119 (34%) |  |
| 4 | 41 (25%) |  | 45 (21%) |  | | 77 (26%) |  | 88 (25%) |  |
| 5 | 14 (9%) |  | 17 (8%) |  | | 33 (11%) |  | 22 (6%) |  |
| 6 | 5 (3%) |  | 6 (3%) |  | | 12 (4%) |  | 11 (3%) |  |
| Eplet mismatch | 27 (19, 38) | - | 28 (19, 35) | - | | 29 (20, 39) | 1 (<1%) | 27 (19, 38) | - |
| Observed follow-up, years | 4.5 (1.5, 6.8) | - | 4.0 (2.0, 6.9) | - | | 4.3 (2.1, 6.9) | - | 4.4  (1.6, 6.9) | - |
| Biopsy-confirmed TCMR (full study period) | 18 (11%) | - | 19 (9%) | - | | 28 (9%) | - | 31 (9%) | - |
| Biopsy-confirmed ABMR* (full study period) | 23 (14%) | - | 30 (14%) | - | | 35 (12%) | - | 40 (11%) | - |
| De-novo donor specific antibodies (full study period) | 24 (14%) | - | 20 (10%) | - | | 46 (15%) | - | 38 (11%) | - |
| Class I^†^ | 10 (6%) |  | 8 (4%) |  | | 18 (6%) |  | 15 (4%) |  |
| Class II^†^ | 19 (11%) |  | 14 (7%) |  | | 36 (12%) |  | 29 (8%) |  |
| Observed graft failures  (full study period) | 29 (17%) | - | 28 (13%) | - | | 46 (15%) | - | 57 (16%) | - |
| Observed deaths  (full study period) | 49 (30%) | - | 57 (27%) | - | | 96 (32%) | - | 100 (28%) | - |

DCD = Donation after Circulatory Determination of Death

## Supplementary Table S 2: Transplant characteristics for replication, main analysis and full VTC cohorts.

|  | **Replication subcohort** | | **Main analysis cohort** | | **Full VTC cohort** | |
| --- | --- | --- | --- | --- | --- | --- |
| **Characteristic** | **Measure**  **(n = 942)** | **Missing** | **Measure**  **(n = 1029)** | **Missing** | **Measure**  **(n = 1679)** | **Missing** |
| Recipient age, years | 57 (48, 65) | - | 57 (48, 65) | - | 55 (44, 64) | - |
| Recipient sex |  | - |  | - |  | - |
| Female | 342 (36%) |  | 376 (37%) |  | 600 (36%) |  |
| Male | 600 (64%) |  | 653 (63%) |  | 1079 (64%) |  |
| Dialysis vintage, years | 3.1 (2.0, 4.4) | 1 (<1%) | 3.1 (2.1, 4.5) | 1 (<1%) | 2.8 (1.5, 4.3) | 3 (<1%) |
| First transplantation, % | 768 (81%) | - | 832 (81%) | - | 1386 (83%) | - |
| Living donor | 0 (0%) | - | 0 (0%) | - | 255 (15%) | - |
| Donor age, years | 57 (46, 67) | - | 57 (46, 67) | - | 54 (44, 65) | - |
| Donor sex |  | - |  | - |  | - |
| Female | 423 (45%) |  | 466 (45%) |  | 807 (48%) |  |
| Male | 519 (55%) |  | 563 (55%) |  | 872 (52%) |  |
| Kidney donor profile index | 71 (43, 89) | 7 (<1%) | 71 (43, 90) | 7 (<1%) | 63 (38, 86) | 29 (2%) |
| DCD Donor | 135 (14%) | 1 (<1%) | 144 (14%) | 1 (<1%) | 189 (13%) | 256 (15%)* |
| Cold ischemia time, hours | 11.4 (7.3, 15.2) | - | 11.5 (7.5, 15.3) | - | 10.4 (5.3, 14.6) | 3 (<1%) |
| Panel reactive antibodies, % | 0 (0, 0) | 3 (<1%) | 0 (0, 0) | 3 (<1%) | 0 (0, 0) | 81 (5%) |
| Preformed donor HLA specific antibodies | 123 (13%) | 1 (<1%) | 140 (14%) | 1 (<1%) | 204 (14%) | 259 (15%)* |
| HLA serotype mismatch  (HLA-A, HLA-B, HLA-DR) |  | 3 (<1%) |  | 4 (<1%) |  | 25 (1%) |
| 0 | 56 (6%) |  | 61 (6%) |  | 105 (6%) |  |
| 1 | 58 (6%) |  | 61 (6%) |  | 98 (6%) |  |
| 2 | 195 (21%) |  | 211 (21%) |  | 307 (19%) |  |
| 3 | 296 (32%) |  | 321 (31%) |  | 500 (30%) |  |
| 4 | 227 (24%) |  | 251 (24%) |  | 398 (24%) |  |
| 5 | 77 (8.2%) |  | 86 (8%) |  | 178 (11%) |  |
| 6 | 30 (3.2%) |  | 34 (3%) |  | 68 (4%) |  |
| HLA eplet mismatch | 28 (19, 37) | 1 (<1%) | 28 (19, 38) | 1 (<1%) | 29 (19, 38) | 40 (2%) |
| Observed follow-up, years | 4.6 (2.5, 7.1) | - | 4.3 (1.9, 6.9) | - | 4.5 (2.0, 7.4) | - |
| Biopsy-confirmed rejection (1st year) | 79 (8%) | - | 88 (9%) | - | 163 (10%) | - |
| Observed graft failures (full study period) | 102 (11%) | - | 160 (16%) | - | 251 (15%) | - |
| Observed deaths (full study period) | 255 (27%) | - | 302 (29%) | - | 444 (26%) | - |

* Not relevant and recorded for living donor transplantations.
DCD = Donation after Circulatory Determination of Death

## Supplementary Table S 3: Replication subcohort models.

|  | **nsSNP-tms** | | **nsSNP-tcmsk** | | **Model with composite score** | | |
| --- | --- | --- | --- | --- | --- | --- | --- |
| **Characteristic** | **Hazard ratio  (95% CI)** | **p-value** | **Hazard ratio (95% CI)** | **p-value** |  | **Hazard ratio (95% CI)** | **p-value** |
| **Non-HLA score*** | **1.31 (1.03, 1.67)** | **0.028** | **1.33 (1.02, 1.74)** | **0.033** | **Composite score** | **1.91 (1.11, 3.31)** | **0.020** |
| HLA eplet mismatch* | 1.20 (0.90, 1.60) | 0.208 | 1.19 (0.90, 1.57) | 0.227 |  |  |  |
| KDPI* | 1.57 (1.06, 2.31) | 0.024 | 1.55 (1.04, 2.32) | 0.030 | KDPI* | 1.53 (1.04, 2.27) | 0.033 |
| Donor sex (male) | 1.21 (0.77, 1.88) | 0.402 | 1.21 (0.78, 1.89) | 0.392 | Donor sex (male) | 1.21 (0.78, 1.88) | 0.400 |
| Preformed DSAs (yes) | 1.21 (0.69, 2.10) | 0.509 | 1.18 (0.68, 2.04) | 0.561 | Preformed DSAs (yes) | 1.18 (0.68, 2.05) | 0.559 |
| Cold ischemia time (h) | 1.02 (0.98, 1.06) | 0.279 | 1.02 (0.98, 1.06) | 0.267 | Cold ischemia time (h) | 1.02 (0.98, 1.06) | 0.261 |
| Pi-hat* | 1.16 (0.81, 1.67) | 0.426 | 1.14 (0.80, 1.64) | 0.467 | Pi-hat* | 1.13 (0.79, 1.61) | 0.513 |

* Standardised by interquartile ranges
All coefficients for the main models presented in Table 2 of the main manuscript. Right panel also gives model for composite score of nsSNP-tcmsk and HA eplet mismatch in this subcohort for reference. For all models 8 observations were removed due to missing data; 89 death-censored graft-losses within 7.5 years after transplantation were used in the models. KDPI = Kidney Donor Profile Index; DSA = Donor HLA specific antibody

## Supplementary Table S 4: Main analysis models.

|  | **Model with scores separately** | | **Model with composite score** | | |
| --- | --- | --- | --- | --- | --- |
| **Characteristic** | **Hazard ratio  (95% CI)** | **p-value** |  | **Hazard ratio (95% CI)** | **p-value** |
| **nsSNP-tcmsk*** | **1.19 (0.97, 1.47)** | **0.096** | **Composite score** | **1.76 (1.20, 2.57)** | **0.004** |
| HLA eplet mismatch* | 1.25 (0.99, 1.57) | 0.062 |  |  |  |
| KDPI* | 1.42 (1.05, 1.92) | 0.023 | KDPI* | 1.42 (1.05, 1.92) | 0.023 |
| Donor sex (male) | 1.07 (0.76, 1.49) | 0.699 | Donor sex (male) | 1.07 (0.77, 1.49) | 0.699 |
| Preformed DSAs (yes) | 1.56 (1.04, 2.35) | 0.034 | Preformed DSAs (yes) | 1.55 (1.03, 2.34) | 0.037 |
| Cold ischemia time (h) | 1.03 (1.01, 1.06) | 0.015 | Cold ischemia time (h) | 1.03 (1.01, 1.06) | 0.015 |
| Pi-hat* | 1.05 (0.79, 1.38) | 0.755 | Pi-hat* | 1.05 (0.80, 1.39) | 0.717 |

* Standardised by interquartile ranges
All coefficients for the main models presented in Table 2 of the main manuscript. Model data comprised 1029 donor and recipient pairs. For all models 8 observations were removed due to missing data; 147 death-censored graft-losses within 7.5 years after transplantation were used in the models. KDPI = Kidney Donor Profile Index; DSA = Donor HLA specific antibody

## Supplementary Table S 5: Main analysis model diagnostics - Tests for Schoenfeld residuals.

| **Model with scores separately** | | **Model with composite score** | |
| --- | --- | --- | --- |
| **Characteristic** | **p-value** |  | **p-value** |
| **nsSNP-tcmsk** | 0.42 | **Composite score** | 0.74 |
| HLA eplet mismatch | 0.88 |  |  |
| KDPI | 0.35 | KDPI | 0.36 |
| Donor sex (male) | 0.85 | Donor sex (male) | 0.86 |
| Preformed DSAs (yes) | 0.15 | Preformed DSAs (yes) | 0.14 |
| Cold ischemia time (h) | 0.12 | Cold ischemia time (h) | 0.12 |
| Pi-hat | 0.25 | Pi-hat | 0.25 |
| Global | 0.40 |  | 0.41 |

Table shows result for models in main analysis cohort. Similar results were obtained for the replication subcohort, where no indication for non-proportionality was found either. KDPI = Kidney Donor Profile Index; DSA = Donor HLA specific antibody

## Supplementary Table S 6: Main model for composite score with different administrative censoring times.

| **Censoring time** | **1 Year** | **3 Years** | **5 Years** | **7.5 Years (Main analysis)** |
| --- | --- | --- | --- | --- |
| **Characteristic** | **Hazard ratio  (95% CI)** | **Hazard ratio  (95% CI)** | **Hazard ratio  (95% CI)** | **Hazard ratio  (95% CI)** |
| **Composite score** | **2.04 (1.27, 3.26)** | **1.60 (1.01, 2.50)** | **1.61 (1.07, 2.42)** | **1.76 (1.20, 2.57)** |
| KDPI* | 1.33 (0.88, 2.02) | 1.37 (0.95, 1.98) | 1.40 (1.01, 1.95) | 1.42 (1.05, 1.92) |
| Donor sex (male) | 0.98 (0.62, 1.55) | 1.07 (0.72, 1.61) | 1.13 (0.78, 1.63) | 1.07 (0.77, 1.49) |
| Preformed DSAs (yes) | 1.98 (1.16, 3.35) | 1.69 (1.04, 2.76) | 1.51 (0.96, 2.37) | 1.55 (1.03, 2.34) |
| Cold ischemia time (h) | 1.05 (1.01, 1.08) | 1.04 (1.01, 1.08) | 1.04 (1.01, 1.07) | 1.03 (1.01, 1.06) |
| Pi-hat* | 0.90 (0.60, 1.35) | 0.91 (0.64, 1.29) | 0.97 (0.72, 1.32) | 1.05 (0.80, 1.39) |

* Standardised by interquartile ranges
All coefficients for the main models with outcomes censored administratively at 1, 3 and 5 years after transplantation. For all models 8 observations were removed due to missing data; 78, 101, 127, and 147 death-censored graft-losses within 1, 3, 5, and 7.5 years after transplantation were used in the models, respectively. KDPI = Kidney Donor Profile Index; DSA = Donor HLA specific antibody

## Supplementary Table S 7: Sensitivity analysis removing 67 transplants present in earlier study.

|  | **Model with scores separately** | | **Model with composite score** | | |
| --- | --- | --- | --- | --- | --- |
| **Characteristic** | **Hazard ratio  (95% CI)** | **p-value** |  | **Hazard ratio (95% CI)** | **p-value** |
| **nsSNP-tcmsk*** | **1.15 (0.92, 1.44)** | **0.212** | **Composite score** | **1.70 (1.13, 2.54)** | **0.010** |
| HLA eplet mismatch* | 1.27 (1.00,1.62) | 0.050 |  |  |  |
| KDPI* | 1.43 (1.05, 1.95) | 0.023 | KDPI* | 1.45 (1.06, 1.97) | 0.020 |
| Donor sex (male) | 1.04 (0.73, 1.48) | 0.836 | Donor sex (male) | 1.04 (0.73, .47) | 0.827 |
| Preformed DSAs (yes) | 1.69 (1.10, 2.59) | 0.016 | Preformed DSAs (yes) | 1.68 (1.09, 2.58) | 0.019 |
| Cold ischemia time (h) | 1.03 (1.00, 1.06) | 0.069 | Cold ischemia time (h) | 1.03 (0.99, 1.06) | 0.073 |
| Pi-hat* | 0.98 (0.73, 1.30) | 0.871 | Pi-hat* | 0.99 (0.74, 1.32) | 0.946 |

* Standardised by interquartile ranges
For all models 8 observations were removed due to missing data; 134 death-censored graft-losses within 7.5 years after transplantation were used in the models. KDPI = Kidney Donor Profile Index; DSA = Donor HLA specific antibody

## Supplementary Table S 8: Generalisability of main models to all-cause graft loss in main analysis cohort.

|  | **Model with scores separately** | | **Model with composite score** | | |
| --- | --- | --- | --- | --- | --- |
| **Characteristic** | **Hazard ratio  (95% CI)** | **p-value** |  | **Hazard ratio (95% CI)** | **p-value** |
| **nsSNP-tcmsk*** | **1.08 (0.94, 1.23)** | **0.286** | **Composite score** | **1.47 (1.15, 1.87)** | **0.003** |
| HLA eplet mismatch* | 1.23 (1.06, 1.42) | 0.006 |  |  |  |
| KDPI* | 1.85 (1.51, 2.27) | <0.001 | KDPI* | 1.87 (1.52, 2.29) | <0.001 |
| Donor sex (male) | 1.04 (0.84, 1.28) | 0.736 | Donor sex (male) | 1.04 (0.84, 1.28) | 0.718 |
| Preformed DSAs (yes) | 0.99 (0.72, 1.35) | 0.925 | Preformed DSAs (yes) | 0.97 (0.71, 1.34) | 0.864 |
| Cold ischemia time (h) | 1.01 (0.99, 1.03) | 0.461 | Cold ischemia time (h) | 1.01 (0.99, 1.03) | 0.483 |
| Pi-hat* | 1.04 (0.87, 1.25) | 0.668 | Pi-hat* | 1.06 (0.88, 1.27) | 0.556 |

* Standardised by interquartile ranges
Models based on main analysis cohort; 355 combined recipient deaths or graft losses within 7.5 years after transplantation were used in these models. For both models, 8 observations were removed due to missing data. KDPI = Kidney Donor Profile Index, DSA = Donor HLA specific antibody

## Supplementary Table S 9: Cox proportional hazard model for secondary TCMR and ABMR outcomes in main analysis cohort.

|  | **Model for TCMR** | | **Model for ABMR** | | |
| --- | --- | --- | --- | --- | --- |
| **Characteristic** | **Hazard ratio  (95% CI)** | **p-value** |  | **Hazard ratio (95% CI)** | **p-value** |
| **Composite score** | **1.56 (0.95, 2.58)** | **0.081** | **Composite score** | **1.40 (0.91, 2.17)** | **0.128** |
| KDPI* | 1.25 (0.85, 1.85) | 0.255 | KDPI* | 1.01 (0.75, 1.38) | 0.968 |
| Donor sex (male) | 0.93 (0.60, 1.44) | 0.752 | Donor sex (male) | 0.99 (0.69, 1.43) | 0.968 |
| Preformed DSAs (yes) | 1.00 (0.56, 1.80) | 0.992 | Preformed DSAs (yes) | 3.63 (2.53, 5.21) | <0.001 |
| Cold ischemia time (h) | 1.04 (1.00, 1.08) | 0.031 | Cold ischemia time (h) | 1.02 (0.99, 1.05) | 0.227 |
| Pi-hat* | 0.88 (0.60, 1.29) | 0.506 | Pi-hat* | 0.92 (0.67, 1.27) | 0.628 |

* Standardised by interquartile ranges
Models based on main analysis cohort; 95 TCMRs and 124 ABMRs (including 55 microvascular inflammations) within 7.5 years after transplantation were used in these models, respectively. For both models, 8 observations were removed due to missing data. KDPI = Kidney Donor Profile Index; DSA = Donor HLA specific antibody

## Supplementary Table S 10: Main model including time-dependent covariate for the formation of de-novo DSAs.

|  | **Model with scores separately** | | **Model with composite score** | | |
| --- | --- | --- | --- | --- | --- |
| **Characteristic** | **Hazard ratio  (95% CI)** | **p-value** |  | **Hazard ratio (95% CI)** | **p-value** |
| **nsSNP-tcmsk*** | **1.17 (0.95, 1.44)** | **0.152** | **Composite score** | **1.56 (1.06, 2.31)** | **0.026** |
| HLA eplet mismatch* | 1.16 (0.91, 1.48) | 0.218 |  |  |  |
| dnDSAs (yes) | 3.37 (2.06, 5.52) | <0.001 | dnDSAs (yes) | 3.32 (2.04, 5.43) | <0.001 |
| KDPI* | 1.40 (1.04, 1.90) | 0.029 | KDPI* | 1.40 (1.04, 1.90) | 0.028 |
| Preformed DSAs (yes) | 1.50 (0.98, 2.27) | 0.059 | Preformed DSAs (yes) | 1.50 (0.98, 2.28) | 0.059 |
| Donor sex (male) | 1.12 (0.79, 1.57) | 0.528 | Donor sex (male) | 1.11 (0.79, 1.56) | 0.533 |
| Cold ischemia time (h) | 1.03 (1.01, 1.06) | 0.015 | Cold ischemia time (h) | 1.03 (1.01, 1.06) | 0.014 |
| Pi-hat* | 1.03 (0.79, 1.36) | 0.813 | Pi-hat* | 1.04 (0.79, 1.36) | 0.794 |

* Standardised by interquartile ranges
Model data comprised 1148 observations from 1029 donor and recipient pairs; 147 death-censored graft losses within 7.5 years after transplantation were used in these models. For both models, 9 observations were removed due to missing data. KDPI = Kidney Donor Profile Index; DSA = Donor HLA specific antibody, dnDSA = de-novo DSA

## Supplementary Table S 11: Restricted mean survival times for strata defined by median scores of nsSNP-tcmsk and eplet mismatch in main analysis cohort.

| **Year** | **Neither score lower than median** | **Both scores lower than median** | **Difference** |
| --- | --- | --- | --- |
| 1 | 0.91 (0.88, 0.94) | 0.96 (0.94, 0.97) | 0.05 (0.01, 0.08) |
| 3 | 2.65 (2.55, 2.75) | 2.82 (2.76, 2.89) | 0.17 (0.06, 0.29) |
| 5 | 4.31 (4.12, 4.50) | 4.65 (4.53, 4.77) | 0.34 (0.12, 0.56) |
| 7 | 5.84 (5.54, 6.14) | 6.41 (6.21, 6.61) | 0.57 (0.20, 0.93) |

Restricted mean survival times are given by point estimates (95% confidence intervals derived by bootstrap) and represent times in years. Strata are defined by the median scores of nsSNP-tcmsk and eplet mismatch (high mismatch given when neither score is lower than their respective medians, low mismatch when both scores are lower than their respective medians). The values were estimated using direct regression standardisation with a Cox proportional hazards model adjusted for donor age, KDPI, donor sex, presence of preformed DSAs, cold ischemia time and Pi-hat. The adjusted curves themselves are shown in Supplementary Figure S 9.

## Supplementary Table S 12: Generalisability of main model to cohort including transplant pairs with non-European ancestry.

|  | **Model with scores separately** | | **Model with composite score** | | |
| --- | --- | --- | --- | --- | --- |
| **Characteristic** | **Hazard ratio  (95% CI)** | **p-value** |  | **Hazard ratio (95% CI)** | **p-value** |
| **nsSNP-tcmsk*** | **1.12 (1.02, 1.24)** | **0.024** | **Composite score** | **1.72 (1.22, 2.41)** | **0.002** |
| HLA eplet mismatch* | 1.22 (1.00, 1.50) | 0.049 |  |  |  |
| KDPI* | 1.65 (1.24, 2.21) | <0.001 | KDPI* | 1.65 (1.24, 2.19) | <0.001 |
| Donor sex (male) | 1.03 (0.75, 1.40) | 0.872 | Donor sex (male) | 1.03 (0.75, 1.40) | 0.870 |
| Preformed DSAs (yes) | 1.68 (1.15, 2.47) | 0.008 | Preformed DSAs (yes) | 1.67 (1.13, 2.45) | 0.009 |
| Cold ischemia time (h) | 1.04 (1.01, 1.07) | 0.003 | Cold ischemia time (h) | 1.04 (1.01, 1.07) | 0.003 |
| Pi-hat* | 1.07 (0.98, 1.17) | 0.119 | Pi-hat* | 1.08 (0.99, 1.19) | 0.084 |

* Standardised by interquartile ranges
Model data comprised 1174 donor and recipient pairs of whom either member could be of non-European genetic ancestry; 171 death-censored graft losses within 7.5 years after transplantation were used in these models. For both models, 13 observations were removed due to missing data. KDPI = Kidney Donor Profile Index; DSA = Donor HLA specific antibody

## Supplementary Table S 13: Sensitivity analysis for restricted mean survival time through alternative computation based on pseudo-values in main analysis cohort.

| **Year** | **High mismatch** | **Low mismatch** | **Difference** |
| --- | --- | --- | --- |
| 1 | 0.92 | 0.97 | 0.05 (0.01, 0.09) |
| 3 | 2.68 | 2.87 | 0.19 (0.06, 0.32) |
| 5 | 4.37 | 4.71 | 0.34 (0.10, 0.57) |
| 7 | 5.94 | 6.45 | 0.51 (0.15, 0.88) |

# Supplementary Figures

## Supplementary Figure S 1: Flowchart of genomic variant inclusion based on our main analysis cohort.

**Supplementary Figure S 1: Flowchart of genomic variant inclusion based on our main analysis cohort.** Top most panel refers to all reviewed human proteins entries in UniProt. “Proteins” refers to the number of unique protein entries in the UniProtKB (<https://www.uniprot.org>), “Transcripts” refers to the number of unique Ensembl stable identifiers linked to those UniProt entries, and “Mismatched transcripts” refers to the number of those unique “Transcripts” for which at least one mismatch was identified in our main analysis cohort. The full list of 6904 final transcripts used is provided as additional supplementary file.

## Supplementary Figure S 2: Distributions of mismatch scores in main analysis cohort.

**Supplementary Figure S 2: Distributions of mismatch scores in main analysis cohort.** In all panels the continuous distributions of the HA and non-HLA mismatch scores studied in the main analysis cohort are displayed by densities and boxplots. Individual data points are jittered to avoid overlap. See the Methods section for details on the definitions and the computation of the scores.

## Supplementary Figure S 3: Scatter plots of HLA eplet mismatch and non-HLA mismatch scores in the main analysis cohort.

**Supplementary Figure S 3: Scatter plots of HLA eplet mismatch and non-HLA mismatch scores in the main analysis cohort.** All panels show pairwise scatter plots of the mismatch scores obtained from each donor-recipient pair. See the Methods section for details on the definitions and the computation of the scores.

## Supplementary Figure S 4: Scatter plot of nsSNP-tcmsk mismatch score and genomic distance (Pi-hat, Identity by descent) in the main analysis cohort.

**Supplementary Figure S 4: Scatter plot of nsSNP-tcmsk mismatch score and genomic distance (Pi-hat, Identity by descent) in the main analysis cohort.** See the Methods section for details on the definitions and the computation of nsSNP-tcmsk and Pi-hat.

## Supplementary Figure S 5: Main model diagnostics - Plots for Schoenfeld residuals.

**Supplementary Figure S 5: Main model diagnostics - Plots for Schoenfeld residuals.** Schoenfeld residuals for the main Cox proportional hazards models fitted in the main analysis cohort. The upper set of panels show the residuals for all variables in the model comprising HLA eplets mismatch and nsSNP-tcmsk separately, the lower set of panels show the residuals for all variables in the model comprising the composite score. In addition to the residuals themselves, a smoothing line using a LOESS smoother including 95% confidence interval is shown in red. The x-axis was transformed using the Kaplan-Meier estimate of the survival function as per the recommendations in the literature.

## Supplementary Figure S 6: Assessment of the linearity assumption for mismatch scores using penalised splines.

**Supplementary Figure S 6: Assessment of the linearity assumption for mismatch scores using penalised splines.** Plots shows log hazard ratios (relative to median of mismatch scores) from Cox models as in Table 2 of the main manuscript (top row representing model using both HLA eplet mismatch and nsSNP-tcmsk with spline terms, bottom row for model with composite score), further adjusted for KDPI, presence of preformed DSAs, donor sex, cold ischemia time and Pi-hat. HLA eplet mismatch, nsSNP-tcmsk and the composite score were modelled using penalised splines with 4 degrees of freedom. Likelihood ratio tests comparing a model with the use of a spline transformed variable and without resulted in non-significant p-values for all plots shown above, indicating no strong evidence for the presence of non-linear effects. The x-axis of the plot is truncated at the 10% and 90% quantiles of the mismatch scores, as outside of this range the 95% confidence intervals for the splines (shown by dotted lines in red) become very wide due to sparse data. Note further that the main goal of this analysis was to check the plausibility of the linearity assumption for the mismatch scores - the robust estimation of a non-linear effect using splines was not the goal, as the event number was quite limited for that purpose.

## Supplementary Figure S 7: Cumulative incidence functions for graft loss and competing event death in the main analysis cohort.

**Supplementary Figure S 7: Cumulative incidence functions for graft loss and competing event death in the main analysis cohort.** The upper panel shows the cumulative incidences for our primary outcome of death censored graft loss (dotted line) and the competing event recipient death (solid line). The lower panel shows the number of recipients at risk for any event at a specific point in time, and the number of events of any type up to a given point in time. The cumulative incidences were computed using the R package “tidycmprsk” version 1.1.0.

## Supplementary Figure S 8: Univariable Kaplan-Meier plots of TCMR and ABMR after transplantation stratified by quartiles of the composite score in the main analysis cohort.

**Supplementary Figure S 8: Univariable Kaplan-Meier plots of TCMR and ABMR after transplantation stratified by quartiles of the composite score in the main analysis cohort.** Plots show survival curves stratified by quartiles of the composite score of HLA eplet mismatch and nsSNP-tcmsk, merging the middle 2^nd^ and 3^rd^ quartiles. The curves comprise 96 TCMRs and 128 ABMRs (55 of the 128 ABMRS were categorised as microvascular inflammations), respectively.

## **Supplementary Figure S 9:** Univariable **Kaplan-Meier and adjusted survival curves for death-censored graft survival using the combination of HLA eplet mismatch and nsSNP-tcmsk by median strata in the main analysis cohort.**

**Supplementary Figure S 9: Univariable Kaplan-Meier and adjusted survival curves for death-censored graft survival using the combination of HLA eplet mismatch and nsSNP-tcmsk by median strata in the main analysis cohort.** Recreation of Figure 3 in main manuscript using a simplified method to combine HLA eplet mismatch and nsSNP-tcmsk. Left panel shows univariable Kaplan-Meier survival curves, right panel shows adjusted survival curves. Here the stratification is defined through the medians of both scores. “Both” refers to those D/R pairs for whom both HLA eplet mismatch and nsSNP-tcmsk score are lower than their respective medians (i.e. low overall mismatch), “Neither” refers to pairs for whom both scores are higher than their respective medians (i.e. high overall mismatch). “Only one” means pairs for whom one of the scores is lower, and the other is higher than the respective median. The resulting RMSTs are given in Supplementary Table S 1110.

## Supplementary Figure S 10: Assessment of stability of hazard ratio estimate of composite score via bootstrap in the main analysis cohort.

**Supplementary Figure S 10: Assessment of stability of hazard ratio estimate of composite score via bootstrap in the main analysis cohort.** Plot shows the bootstrap distribution of hazard ratios for the composite score, i.e. the distribution obtained when resampling the main analysis cohort 500 times with replacement, and computing the main Cox model in the resampled dataset. The dashed line indicates the median of the slightly right skewed bootstrap distribution, the red line indicates the estimate from our main model in Table 2 (1.76); both lines overlap, indicating good stability of the estimate. Further, almost all bootstrap samples led to an estimated hazard ratio > 1 (i.e. 99.8%), which would be another way to provide a p-value for the association of the composite score and is in good agreement with the main model result.
