# Supplementary material for: The combined impact of HLA and non-HLA mismatch between donors and recipients on kidney transplant survival: a genomic analysis in a prospective cohort
Source: eBioMedicine. 2026 Aug 5;131:106420. doi: 10.1016/j.ebiom.2026.106420 (PMC13471318; doi:10.1016/j.ebiom.2026.106420)
Supplement: Supplementary file 2 [file mmc3.docx]

**International Genetics & Translational Research in Transplantation Network**

| **First names** | **Surname** |
| --- | --- |
| Brian | Piening |
| Alexa | Dowdell |
| Mario | Deng |
| Martin | Cadeiras |
| Mengqi | Zhang |
| Matthew B. | Lanktree |
| Krzysztof | Kiryluk |
| Francesca | Zanoni |
| Sangho | Lee |
| Ajay | Israni |
| Brendan | Keating |
| Rainer | Oberbauer |
| Goran B. | Klintmalm |
| Jacqueline | O’Leary |
| Kim M. | Olthoff |
| Elizabeth | Rand |
| Abraham | Shaked |
| John A. | Belperio |
| Marc | Buijsrogge |
| Jason D. | Christie |
| Paul | Corris |
| Andrew | Fisher |
| Allan R. | Glanville |
| Samuel | Goldfarb |
| David | Lederer |
| Keith C. | Meyer |
| Henny G. | Otten |
| Scott M. | Palmer |
| David | Wilkes |
| Nathan | Pankratz |
| Pam | Jacobson |
| Loren | Gragert |
